# Combined Use of Emodin and Ginsenoside Rb1 Exerts Synergistic Neuroprotection in Cerebral Ischemia/Reperfusion Rats

**DOI:** 10.3389/fphar.2018.00943

**Published:** 2018-08-28

**Authors:** Yan Li, Qing-qing Xu, Chun-shuo Shan, Yi-hua Shi, Yong Wang, Guo-qing Zheng

**Affiliations:** Department of Neurology, The Second Affiliated Hospital and Yuying Children’s Hospital of Wenzhou Medical University, Wenzhou, China

**Keywords:** ginsenoside Rb1, emodin, cerebral ischemia/reperfusion, Connexin 43, Aquaporin 4

## Abstract

Acute ischemic stroke (AIS) generally causes neurological dysfunction and poses a serious threat to public health. Here, we aimed to assess the independent and combined effects of ginsenoside Rb1 (GRb1) and Emodin on neuroprotection through regulating Connexin 43 (Cx43) and Aquaporin 4 (AQP4) expression in cerebral ischemia/reperfusion (I/R) model rats. Adult male Sprague-Dawley (SD) rats were randomly divided into five groups: sham group, I/R group, Emodin group, GRb1 group and Emodin+GRb1 group. They were further allocated to four subgroups according to the 6h, 1d, 3d, and 7d time points except the sham group. Based on the modified Longa suture method, the focal cerebral I/R model was established by middle cerebral artery occlusion (MCAO). The neurological deficit scores (NDS), blood brain barrier (BBB) permeability and cerebral infarction area were assessed at each corresponding time point. Cx43 and AQP4 levels were assessed by Real-time PCR and Immunofluorescence. Compared with I/R group, both the independent and combined use of GRb1 and Emodin could alleviate NDS, reduce the BBB permeability, reduce the infarction area and down-regulate Cx43 and AQP4 expression at 6h, 1d, 3d, and 7d after I/R (*P* < 0.05). The Emodin+GRb1 group had more significant effects than Emodin group and GRb1 group (*P* < 0.05). In conclusion, the combination of Emodin and GRb1 exerts synergistically neuroprotective functions through regulating AQP4 and Cx43 after I/R.

## Introduction

Acute ischemic stroke (AIS) generally causes vascular occlusions and cerebral blood flow blocked, brain ischemia and hypoxia, and necrosis of brain tissue, which finally results in neurological dysfunction (Bangalore et al., 2014), accounting for approximately 87% of strokes (Mozaffarian et al., 2016). There are 15 million new incidence cases of ischemic stroke and 6.5 million ischemic stroke-related deaths per year, and by 2030 ischemic stroke will cause the annual loss of over 200 million disability-adjusted life years globally (Neuhaus et al., 2017). According to the 2018 guideline from the American Heart Association/American Stroke Association (AHA/ASA), intravenous thrombolysis of recombinant tissue type plasminogen activator (rtPA) and/or mechanical thrombectomy are two effective treatments for AIS patients (Powers et al., 2018). However, rtPA is only applied in less than 10% AIS patients (Shobha et al., 2011) and the recanalisation rate is not ideal (approximately 46%) (Rha and Saver, 2007). Furthermore, the narrow therapeutic time window of 4.5 h post ischemic injury and serious complications such as fatal intracerebral hemorrhage hinder its clinical use (Jauch et al., 2013). As for mechanical thrombolysis, the requirements of rapid cerebral angiography in experienced stroke centers and qualified neurointerventional physicians largely impose restrictions on its clinical use (Moussaddy et al., 2018). In addition, most patients treated with thrombolytic therapy are still found to have neurological dysfunction after treatments (Moussaddy et al., 2018). Thus, it is particularly necessary to seek alternative therapeutic approaches for AIS patients.

Chinese herbal medicine (CHM), as a pharmacological form of traditional Chinese medicine (TCM), has been used for stroke from thousands of years ago and now is still used worldwide (Wu et al., 2007). Ginseng, first recorded in Shennong Bencaojing (The Divine Farmer’s Herb-Root Classic) in 196 AD, is one of the most widely used traditional CHM in the world (Murthy et al., 2014). Ginsenoside Rb1 (GRb1) is an effective ingredient of ginseng. GRb1 can exert extensive bioactivity in the central nervous system (CNS), especially playing a predominant role in neuroprotection in ischemic stroke (Vaibhav et al., 2014; Chen et al., 2015). Furthermore, Emodin, an anthraquinone derivative mainly from the rhizome of Rheum palmatum L., is the main active monomer of Da Huang (Radix et Rhizoma Rhei), which was found to have potentially neuroprotective effects on cerebral ischemia (Liu et al., 2015). AIS involves complex pathologic processes (Brouns and De Deyn, 2009), and thus intervention of multiple targets is necessary. Herb pair refers to two relatively fixed herbs that used in unique combination in TCM clinic, which is the simplest and the most fundamental form of multi-herb therapy to specifically achieve mutual promotion (Wang et al., 2012). Ginseng and Da Huang belong to famous herb pairs that are commonly used couplet drugs for stroke. As the ingredients of herb compatibility are various and complicated, the compatibility of bioactive components can be more scientific to clarify the compatibility theory (Lu et al., 2014). Administration of herb pairs and their component compatibility after cerebral ischemia/reperfusion (I/R) injury were found effective for ischemic stroke (Han et al., 2017). GRb1 played a predominate role on neuroprotection by protecting blood-brain barrier (BBB) integrity (Chen et al., 2015), increasing the regional cerebral blood flow (rCBF) and the stability of neuronal ultrastructure (Wang et al., 2017) and alleviating the morphological lesion concomitant with improvement of cognitive and sensorimotor deficits (Yan et al., 2018). Emodin had a significantly neuroprotective effect via anti-apoptosis (Ahn et al., 2016) and anti-inflammation (Li et al., 2005; Wu et al., 2009), subsequently enhancing behavioral function in cerebral ischemia. However, whether the combined effects of GRb1 and Emodin have addictive pharmacological efficacy than that of individual use is still unclear. Thus, we aimed to assess the combined effects of Emodin and GRb1 on neuroprotection in cerebral I/R rats.

Gap junctions (GJ) are clusters of unique membrane channels that play an essential part inn intercellular communication, permitting direct exchange of small molecules and ions between adjacent cells. Connexin (Cx) is a kind of membrane protein that forms the basic structure and function of intercellular GJ (Belousov et al., 2017). As the most extensively expressed Cx in the brain, Connexin 43 (Cx43) plays an important role in the regulation of CNS injury and is widely distributed in astrocytes and vascular endothelial cells in CNS (Söhl and Willecke, 2004; Jeanson et al., 2016). Cx43 can regulate intercellular material exchange and electric signal transmission through the gap junction communication (GJC) and help endothelial cells coupling, which contributes to regulating the growth differentiation and physiological function of nerve cells, and maintaining microenvironmental balance (Chang et al., 2000; Belousov et al., 2017). After I/R injury, the transcription and expression of Cx43 in astrocytes increased and apoptosis information could be mediated by Cx43 to transferred from ischemic central area to the adjacent ischemic penumbra through astrocytic GJC, leading to neuronal cell death and further increasing the infarction area (Le et al., 2014; Gilleron et al., 2018). Thus, inhibiting Cx43 may have neuroprotective effects. Aquaporin (AQP) is of great significance to cerebral water balance regulation and the clinical treatment of neurological diseases (Zador et al., 2007). Aquaporin 4 (AQP4) mainly expressed in astrocytes, ependymal cells, soft meninges, choroid plexus, and the nucleus of the lower mound in the brain (Nielsen et al., 1997; Papadopoulos and Verkman, 2013). AQP4 played an essential part in the formation and dissipation of cerebral edema in the early ischemic stroke (Yao et al., 2015a). AQP4 gene knockout mice experiments also proved that AQP4 loss reduced the cytotoxic edema caused by ischemic stroke (Yao et al., 2015b). It was found that Cx43 and AQP4 were interrelated in water balance regulation and GJC, and the high expression of Cx43 could be decreased with the silencing of AQP4 gene (Kong et al., 2008). Thus, we explore the mechanisms of combined effect of Emodin and GRb1 on Cx43 and AQP4 expression in focal cerebral I/R injury in rats.

## Materials and Methods

### Ethics Statement

All animal experiments abided by the Guide for the Care and Use of Laboratory Animals issued by the United States National Institutes of Health (Publication No. 85-23). Approval for the experimental protocols were approved and regulated by the local ethic committee of the Wenzhou Medical University for animal research (No., wydw2015-0148). All the animals were sacrificed by using anesthesia at the end of the experiment. The utmost possible efforts were made to reduce the suffering of the experimental animals and the number of animals needed for this study.

### Animals, Grouping, and Induction of I/R Model

Adult male Sprague-Daweley (SD) rats (250–280 g) were purchased from Shanghai Laboratory Animal Center (NO., SCXK, Shanghai, 2010-0002). All rats were housed under controlled conditions: 12 h light/dark cycle, temperature 23 ± 2°C, and humidity 50%. These SD rats were provided with free access to both food and water. Rats were randomly divided into five groups: Sham group, I/R group, Emodin group, GRb1 group and Emodin+GRb1 group. Except the sham group, other groups were further divided into 6h, 1d, 3d, and 7d time-point subgroups. The model of focal brain I/R was established according to the modified Longa suture method as our previously published (Gu et al., 2012). SD rats were anaesthetized with 10% chloral hydrate intraperitoneal injection (3 ml/kg). After a midline skin incision, external carotid artery, internal carotid artery (ICA) and pterygopalatine artery of the ICA were exposed, a piece of 3/0 monofilament nylon suture, with its tip rounded by gentle heating, was introduced via lumen of left external carotid artery stump and left ICA to embed into left anterior cerebral artery so that left middle cerebral artery was occluded at its origin. After 2 h of ischemia, the intra-luminal suture was withdrawn from left anterior cerebral artery and right ICA to permit reperfusion. After operation, rats were transferred to electric blanket until animals woke up completely. All rats were allowed free access to food and water. For the Sham group, the same surgical procedures were performed but without occlusion of the MCA.

### Time-Dependence of G-Rb1, Emodin and Emodin+GRb1 Treatment

The GRb1 and emodin used in this study were of high purity (98%) as determined by high performance liquid chromatography. GRb1 powder (1 g) and emodin powder (0.625 g) were dissolved in normal saline (NS) (100 ml), respectively, at the final concentration of 10 mg/ml and 6.25 mg/ml, respectively. Three days before the operation, GRb1 and emodin were given to Emodin group, GRb1 group and Emodin+GRb1 group by intraperitoneal injection at a dose of 40 g kg^-1^ d^-1^ and 25 g kg^-1^ d^-1^, respectively, once a day until the rats were sacrificed. Other groups were given the same volume of NS.

### Neurological Deficit Scores

Neurological deficit scores (NDS) was blindly examined at 6h, 1d, 3d, and 7d after I/R according to the five-point scale described previously by Longa et al. (1989) as follows: score 0, no neurological deficit; score 1, mild focal neurological deficit (with contralateral forelimb flexion); score 2, moderate focal neurological deficit (circling to the contralateral side); score 3, severe focal neurological deficit (falling to the contralateral side); score 4, no spontaneous activity with a depressed level of consciousness or death. Rats with higher score indicated more severity of neurological deficits. Only the rats with score of 1–3 at 2 h after I/R were considered successful models and included in the current study.

### Measurement of BBB Permeability

Extravasation of Evans Blue (EB) in the brain tissue was used to assess the BBB permeability. After the rats were anesthetized, 2% EB dye was injected intravenously 90 min before the given time point into the femoral vein with at a dose of 4 ml kg^-1^. At the given time point after I/R the rats were deeply anesthetized and perfused with 250 ml NS through the left ventricle until the drainage fluid from the atrium became colorless and then the brains were removed and dissected. Each hemisphere was weighed, incubated in methanamide (3 ml) and then heated in 60°C water for 24 h. Samples were then centrifuged for 20 min at 5000 rpm followed by 10 min at 10,000 rpm. The absorbance of the supernatant was measured at 632 nm wavelength using spectrophotometer.

### Triphenyltetrazolium Chloride (TTC) Staining

At 6h, 1d, 3d, and 7d after reperfusion, the rats were rapidly sacrificed and the brains were rapidly removed and coronally sliced into 2 mm thickness. Brain slices were immersed in 2% TTC saline solution and incubated for 15 min at 37°C. Then brain slices were fixed in 10% formalin solution. The infarct region appeared in white color while the normal brain tissue appeared in red. The sections were photographed with a high-resolution camera. The infarction area of each section was quantified by the change in coloration. Infarct size was determined by digital planimetry of the slices using Image-Pro software (Image-Pro Plus 6.0) (Media Cybernetics Inc.).

### *In situ* Immunofluorescent Detection of Cx43 and AQP4 in Brains

Frozen coronal sections were used for detection of *Cx43 and AQP4* with immunofluorescent. The sections were treated with buffer containing 0.2% Triton (Sigma) and 50 mM phosphate-buffered saline (PBS) for three times and 5 min each time, antigen retrieval buffer for three times and 5 min each time, 10% donkey serum for 1 h, and then polyclonal Cx43 (1:400; Abcam) or polyclonal *AQP4* (1:400; Abcam) overnight at 4°C. After rinsed with PBS, the slides were incubated with DyLight 594 donkey anti-mouse secondary antibody (1:400; EarthOx), a kind of fluorophore-labeled donkey anti-mouse IgG (H+L) antibodies, for 1 h at 37°C. Sections were mounted with anti-fade mounting medium (Beyotime). Images were acquired using fluorescent microscope (Nikon) at a constant exposure.

### Real-Time PCR of Cx43 and AQP4 in Brains

Rats were sacrificed at the given time points and were transcardially perfused with NS. The cortex, hippocampus and striatum were isolated from the ischemic regions. Total RNA was extracted using TRIzol (Invitrogen) and then Labeled cDNA was reverse transcribed from the total RNA samples using the Prime Script TM RT reagent Kit (TAKARA). One-step quantitative real-time RT-PCR was performed using a Light Cycler thermal cycler system (Bio-Rad). Quantitative polymerase chain reaction was performed using SYBR Premix Ex Taq II (Takara) and gene-specific primers for 40 cycles according to the manufacturer’s instructions. The primers used were as follows: AQP4: forward, 5′-CATGGAGGTGGAGGACAACC-3′, and reverse, 5′-GCAGGAAATCTGAGGCCAGT-3′, at a fragment length of 200 bp and a temperature of 60°C; GJA1: forward, 5′-GGAAAGTACCAAACAGCAGCAG-3′, and reverse, 5′-CTGGGCACCTCTCTTTCACTT-3′, at a fragment length of 152 bp and a temperature of 60°C; glyceraldehyde-3-phosphate dehydrogenase 2 (GAPDH2): forward, 5′-TGAAGAACAGGGAAGCAGCAA-3′, and reverse, 5′-ATCCAGTCCATTTTCCACCACA-3′, at a fragment length of 200 bp and a temperature of 60°C.

### Statistics Analysis

Data except for the NDSs were expressed as means ± standard (mean ± SD) deviation. Statistical analysis was performed using SPSS 15.0 statistical software. For multiple group experiments, comparisons were made via one-way analysis of variance (ANOVA) and differences between two groups within the multiple groups were analyzed using Dunnett test. NDSs were presented as the medians (ranges) and were analyzed using the Mann–Whitney U test. The significance level was set at *p* < 0.05.

## Results

### Improvement of Neurological Function

**FIGURE 1:**
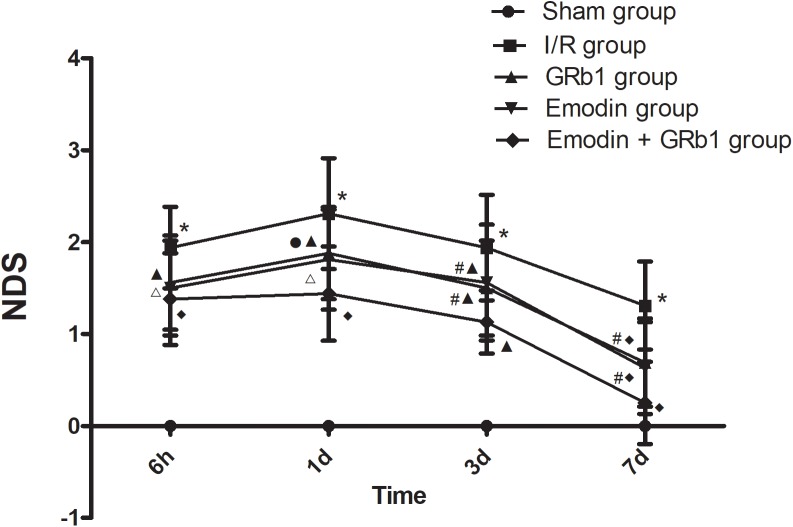
The neurological deficits in different groups after I/R in rats (mean ± SD, *n* = 20). NDS in Sham group, I/R group, GRb1 group, Emodin group, and Emodin+GRb1 group at 6h, 1d, 3d, and 7d. Kruskal–Wallis test followed by the Mann–Whitney U test. ^∗^*p* < 0.001, compared with Sham group. ^▲^*p* < 0.05, ^△^ *p* < 0.01, ^♦^*p* < 0.001, compared with I/R group. ^#^*P* < 0.05, ^●^*p* < 0.01, compared with the Emodin+GRb1 group.

Cerebral infarction resulted in neurological function deficit in the rats, which was mainly detected as contralateral forelimb paralysis. The NDS was evaluated using Zea-Longa criteria. A higher score represents more severe dysfunction. In the present study, the NDS at 6h, 1d, 3d, and 7d after the I/R was evaluated. No neurological deficit was detected in the Sham group. One-way ANOVA showed a significantly difference among Sham group, I/R group, GRb1 group, Emodin group and GRb1+Emodin group in NDS at 6 h (*F* = 56.52, df = 4, *P* < 0.0001), 1d (*F* = 67.07, df = 4, *P* < 0.0001), 3d (*F* = 49.78, df = 4, *P* < 0.0001), and 7d (*F* = 27.22, df = 4, *P* < 0.0001). NDS increased at 6 h after I/R and peaked at 1 d, then decreased gradually but still remained higher than Sham group at 7d. Compared with I/R group, Emodin group, GRb1 group, and Emodin+GRb1 group had a significant difference at the time point of 6h, 1d, 3d, and 7d (*P* < 0.05, *P* < 0.01 or *P* < 0.001). Compared with GRb1 group, Emodin+GRb1 group had a significant difference at 1d, 3d, and 7d after I/R (*P* < 0.05 or *P* < 0.01); Compared with Emodin group, Emodin+GRb1 group had a significant difference at 3d and 7d after I/R (*P* < 0.05). Compared with sham group, I/R group had a significant difference at 6h, 1d, 3d, and 7d (*P* < 0.001) (**Figure 1**).

### Reduction of BBB Disruption

**FIGURE 2:**
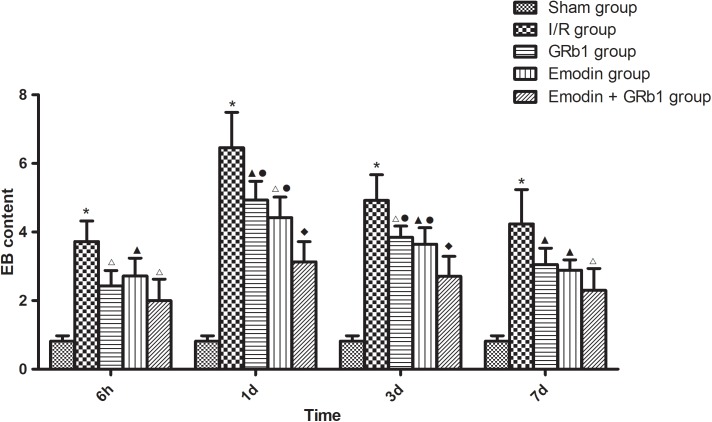
The EB content in different groups after I/R in rats (mean ± SD, *n* = 5). Evans blue leakage experiments for determining BBB permeability after I/R in rats in four sub-groups at 6 h, 1d, 3d, and 7d. One-way analysis of variance (ANOVA) was used for multiple group experiments comparisons and followed by Dunnett test for comparison of two groups within the multiple groups. ^∗^*p* < 0.001, compared with Sham group. ^▲^*p* < 0.05, ^△^ *p* < 0.01, ^♦^*p* < 0.001, compared with I/R group. ^●^*p* < 0.01, compared with the Emodin+GRb1 group.

The permeability of the BBB was quantitatively evaluated by measuring of leakage of EB. One-way ANOVA showed a significantly difference among the five groups in EB content at 6 h (*F* = 22.42, df = 4, *P* < 0.0001), 1d (*F* = 53.32, df = 4, *P* < 0.0001), 3d (*F* = 47.20, df = 4, *P* < 0.0001), and 7d (*F* = 21.99, df = 4, *P* < 0.0001). EB content in the brain increased at 6 h after I/R and peaked at 1d, then descended gradually but remained higher than Sham group at 7d. Compared with I/R group, Emodin group, GRb1 group, and Emodin+GRb1 group had a significant difference at the time point of 6h, 1d, 3d, and 7d (*P* < 0.05, *P* < 0.01, or *P* < 0.001). Compared with GRb1 group, Emodin+GRb1 group had a significant difference at 1d and 3d after I/R (*P* < 0.01); Compared with Emodin group, Emodin+GRb1 group had a significant difference at 1d and 3d after I/R (*P* < 0.01). Compared with Sham group, I/R group had a significant difference at 6h, 1d, 3d, and 7d (*P* < 0.001) (**Figure 2**).

### Reduction of Infarction Area

**FIGURE 3:**
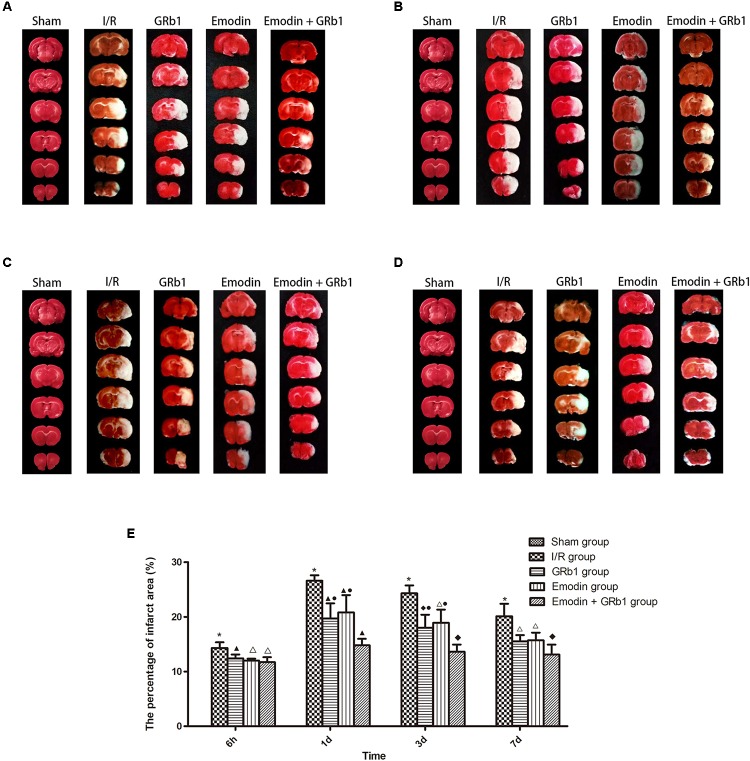
The percentage of infarct area in different groups after I/R in rats (mean ± SD, *n* = 5). TTC staining was used to evaluate cerebral infarction area after I/R in rats in four sub-groups at **(A)** 6h, **(B)** 1d, **(C)** 3d, and **(D)** 7d. **(E)** Quantitative analysis for the results of **(A–D)**. One-way analysis of variance (ANOVA) was used for multiple group experiments comparisons and followed by Dunnett test for comparison of two groups within the multiple groups. ^∗^*p* < 0.001, compared with Sham group. ^▲^*p* < 0.05, ^△^ *p* < 0.01, ^♦^*p* < 0.001, compared with I/R group. ^●^*p* < 0.01, compared with the Emodin+GRb1 group.

One-way ANOVA showed a significantly difference among the five groups in the percentage of infarct area at 6h (*F* = 310.01, df = 4, *P* < 0.0001), 1d (*F* = 126.231, df = 4, *P* < 0.0001), 3d (*F* = 136.82, df = 4, *P* < 0.0001), and 7d (*F* = 122.16, df = 4, *P* < 0.0001). The percentage of infarct area increased at 6 h after I/R and peaked at 1d, then descended gradually but remained higher than Sham group at 7d. Compared with I/R group, Emodin group, GRb1 group, and Emodin+GRb1 group had a significant difference at the time point of 6h, 1d, 3d, and 7d (*P* < 0.05, *P* < 0.01, or *P* < 0.001). Compared with GRb1 group, Emodin+GRb1 group had a significant difference at 1d and 3d after I/R (*P* < 0.01); Compared with Emodin group, Emodin+GRb1 group had a significant difference at 1d and 3d after I/R (*P* < 0.01). Compared with sham group, I/R group had a significant difference at 6h, 1d, 3d, and 7d (*P* < 0.001) (**Figure 3**).

### Decreased Expression of AQP4 and Cx43 With Immunofluorescence Staining

**FIGURE 4:**
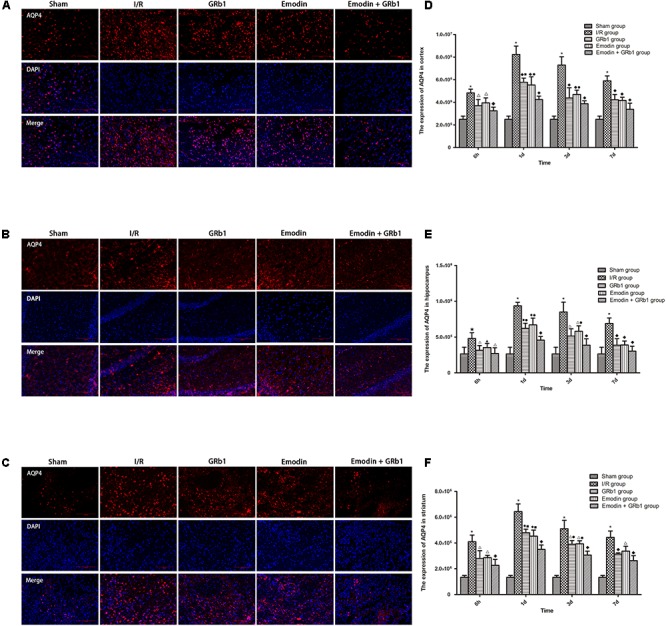
Immunofluorescence staining of AQP4 in I/R rats (mean ± SD, *n* = 5). **(A–C)** The expression of AQP4 in **(A)** cortex, **(B)** hippocampus, and **(C)** striatum with immunofluorescence staining. **(D,E)** Quantitative analysis for the results of **(A–C)**. One-way analysis of variance (ANOVA) was used for multiple group experiments comparisons and followed by Dunnett test for comparison of two groups within the multiple groups. ^★^*p* < 0.01, ^∗^*p* < 0.001, compared with Sham group. ^▲^*p* < 0.05, ^△^ *p* < 0.01, ^♦^*p* < 0.001, compared with I/R group. ^●^*p* < 0.01, ^■^*p* < 0.001, compared with the Emodin+GRb1 group.

One-way ANOVA of AQP4 with the immunofluorescence staining showed a significantly difference among the five groups at 6h (cortex: *F* = 23.88, df = 4, *P* < 0.0001; hippocampus: *F* = 6.57, df = 4, *P* = 0.002; striatum: *F* = 27.99, df = 4, *P* < 0.0001), 1d (cortex: *F* = 79.59, df = 4, *P* < 0.0001; hippocampus: *F* = 55.62, df = 4, *P* < 0.0001; striatum: *F* = 111.55, df = 4, *P* < 0.0001), 3d (cortex: *F* = 40.24, df = 4, *P* < 0.0001; hippocampus: *F* = 23.56, df = 4, *P* < 0.0001; striatum: *F* = 71.21, df = 4, *P* < 0.0001), and 7d (cortex: *F* = 45.63, df = 4, *P* < 0.0001; hippocampus: *F* = 22.91, df = 4, *P* < 0.0001; striatum: *F* = 55.46, df = 4, *P* < 0.0001). The immunofluorescence staining showed that AQP4 increased at 6 h after I/R and peaked at 1d, then descended gradually but remained higher than Sham group at 7d. Compared with I/R group, Emodin group, GRb1 group, and Emodin+GRb1 group had a significant difference at the time point of 6h, 1d, 3d, and 7d (*P* < 0.01 or *P* < 0.001); Compared with GRb1 group, Emodin+GRb1 group had a significant difference at 1d and 3d (striatum) or 1d (cortex and hippocampus) after I/R (*P* < 0.01 or *P* < 0.001); Compared with Emodin group, Emodin+GRb1 group had a significant difference at 1d and 3d after I/R (*P* < 0.01 or *P* < 0.001); Compared with Sham group, I/R group had a significant difference at 6h, 1d, 3d, and 7d (*P* < 0.01 or *P* < 0.001) (**Figure 4**).

**FIGURE 5:**
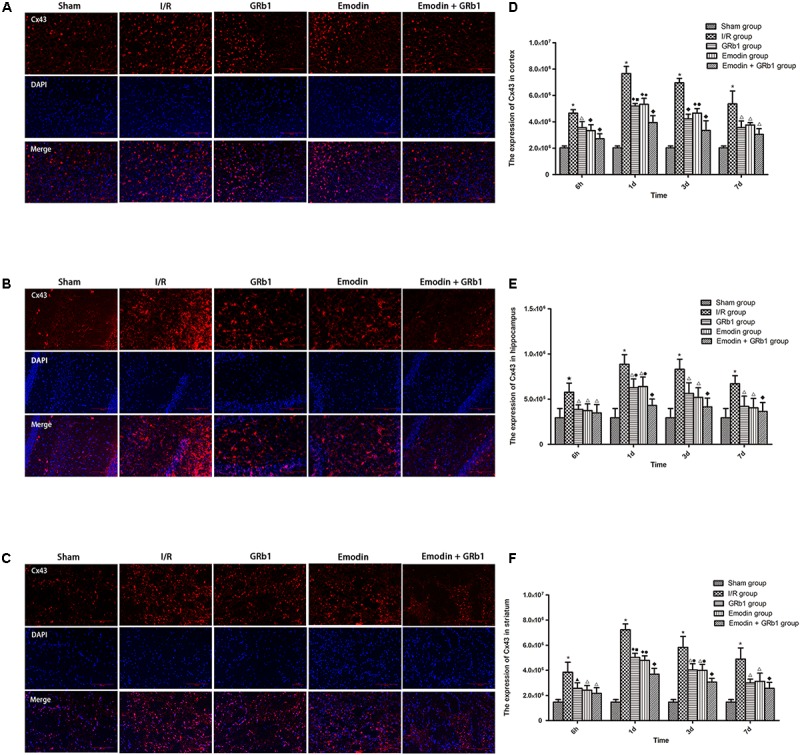
Immunofluorescence staining of Cx43 in I/R rSD, *n* = 5). **(A–C)** The expression of Cx43 in **(A)** cortex, **(B)** hippocampus, and **(C)** striatum with immunofluorescence staining. **(D,E)** Quantitative analysis for the results of **(A–C)**. One-way analysis of variance (ANOVA) was used for multiple group experiments comparisons and followed by Dunnett test for comparison of two groups within the multiple groups. ^★^*p* < 0.01, ^∗^*p* < 0.001, compared with Sham group. ^▲^*p* < 0.05, ^△^ *p* < 0.01, ^♦^*p* < 0.001, compared with I/R group. ^●^*p* < 0.01, ^■^*p* < 0.001, compared with the Emodin+GRb1 group.

One-way ANOVA of Cx43 with the immunofluorescence staining showed a significantly difference among the five groups at 6h (cortex: *F* = 37.69, df = 4, *P* < 0.0001; hippocampus: *F* = 7.73, df = 4, *P* = 0.001; striatum: *F* = 15.09, df = 4, *P* < 0.0001), 1d (cortex: *F* = 128.58, df = 4, *P* < 0.0001; hippocampus: *F* = 27.03, df = 4, *P* < 0.0001; striatum: *F* = 158.18, df = 4, *P* < 0.0001), 3d (cortex: *F* = 90.34, df = 4, *P* < 0.0001; hippocampus: *F* = 17.37, df = 4, *P* < 0.0001; striatum: *F* = 46.05, df = 4, *P* < 0.0001), and 7d (cortex: *F* = 25.53, df = 4, *P* < 0.0001; hippocampus: *F* = 9.75, df = 4, *P* < 0.0001; striatum: *F* = 24.33, df = 4, *P* < 0.0001). Cx43 increased at 6 h after I/R and peaked at 1d, then descended gradually but remained higher than Sham group at 7d. Compared with I/R group, Emodin group, GRb1 group, and Emodin+GRb1 group had a significant difference at the time point of 6h, 1d, 3d, and 7d (*P* < 0.05, *P* < 0.01, or *P* < 0.001); Compared with GRb1 group, Emodin+GRb1 group had a significant difference at 1d and 3d (striatum) or 1d (cortex and hippocampus) after I/R (*P* < 0.01 or *P* < 0.001); Compared with Emodin group, Emodin+GRb1 group had a significant difference at 1d (hippocampus) or 1d and 3d (cortex and striatum) after I/R (*P* < 0.01); Compared with sham group, I/R group had a significant difference at 6h, 1d, 3d, and 7d (*P* < 0.01 or *P* < 0.001) (**Figure 5**).

### Decreased Expression of AQP4 and Cx43 With Real-Time PCR

**FIGURE 6:**
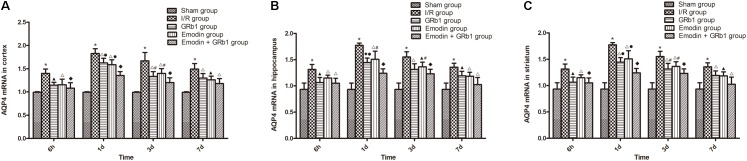
The AQP4 mRNA expression in different groups after I/R in rats (mean ± SD, *n* = 5). **(A)** Cortex; **(B)** hippocampus; **(C)** striatum. Quantitative analysis for the results of groups at 6 h, 24 h, 3d, and 7d, respectively. One-way analysis of variance (ANOVA) was used for multiple group experiments comparisons and followed by Dunnett test for comparison of two groups within the multiple groups. ^∗^*p* < 0.001, compared with Sham group. ^▲^*p* < 0.05, ^△^ *p* < 0.01, ^♦^*p* < 0.001, compared with I/R group. ^●^*p* < 0.01, compared with the Emodin+GRb1 group.

One-way ANOVA of AQP4 with the *Real-time* PCR showed a significantly difference among the five groups at 6 h (cortex: *F* = 14.06, df = 4, *P* < 0.0001; hippocampus: *F* = 10.94, df = 4, *P* < 0.0001; striatum: *F* = 12.99, df = 4, *P* < 0.0001), 1d (cortex: *F* = 64.80, df = 4, *P* < 0.0001; hippocampus: *F* = 44.98, df = 4, *P* < 0.0001; striatum: *F* = 35.64, df = 4, *P* < 0.0001), 3d (cortex: *F* = 23.89, df = 4, *P* < 0.0001; hippocampus: *F* = 24.81, df = 4, *P* < 0.0001; striatum: *F* = 28.00, df = 4, *P* < 0.0001), and 7d (cortex: *F* = 20.19, df = 4, *P* < 0.0001; hippocampus: *F* = 12.94, df = 4, *P* < 0.0001; striatum: *F* = 12.18, df = 4, *P* < 0.0001). With *Real-time PCR*, the results showed that AQP4 increased at 6 h after I/R and peaked at 1d, then descended gradually but remained higher than Sham group at 7d. Compared with I/R group, Emodin group, GRb1 group, and Emodin+GRb1 group had a significant difference at the time point of 6h, 1d, 3d, and 7d (*P* < 0.05, *P* < 0.01, or *P* < 0.001); Compared with GRb1 group, Emodin+GRb1 group had a significant difference at 1d and 3d (cortex and striatum) or 1d (hippocampus) after I/R (*P* < 0.05 or *P* < 0.01); Compared with Emodin group, Emodin+GRb1 group had a significant difference at 1d and 3d after I/R (*P* < 0.05 or *P* < 0.01); Compared with sham group, I/R group had a significant difference at 6h, 1d, 3d, and 7d (*P* < 0.001) (**Figure 6**).

**FIGURE 7:**
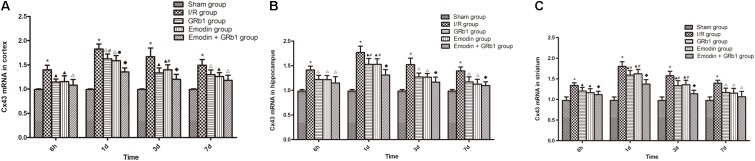
The Cx43 mRNA expression in different groups after I/R in rats (mean ± SD, *n* = 5). **(A)** Cortex; **(B)** hippocampus; **(C)** striatum. Quantitative analysis for the results of groups at 6 h, 24 h, 3d, and 7d, respectively. One-way analysis of variance (ANOVA) was used for multiple group experiments comparisons and followed by Dunnett test for comparison of two groups within the multiple groups. ^∗^*p* < 0.05, compared with Sham group. ^∗^*p* < 0.001, compared with Sham group. ^▲^*p* < 0.05, ^△^ *p* < 0.01, ^♦^*p* < 0.001, compared with I/R group. ^●^*p* < 0.01, compared with the Emodin+GRb1 group.

One-way ANOVA of Cx43 with the *Real-time* PCR showed a significantly difference among the five groups at 6 h (cortex: *F* = 14.56, df = 4, *P* < 0.0001; hippocampus: *F* = 15.76, df = 4, *P* < 0.0001; striatum: *F* = 12.41, df = 4, *P* < 0.0001), 1d (cortex: *F* = 71.43, df = 4, *P* < 0.0001; hippocampus: *F* = 37.98, df = 4, *P* < 0.0001; striatum: *F* = 44.82, df = 4, *P* < 0.0001), 3d (cortex: *F* = 30.38, df = 4, *P* < 0.0001; hippocampus: *F* = 22.92, df = 4, *P* < 0.0001; striatum: *F* = 24.36, df = 4, *P* < 0.0001), and 7d (cortex: *F* = 18.63, df = 4, *P* < 0.0001; hippocampus: *F* = 16.76, df = 4, *P* < 0.0001; striatum: *F* = 11.33, df = 4, *P* < 0.0001). Cx43 increased at 6 h after I/R and peaked at 1d, then descended gradually but remained higher than Sham group at 7d. Compared with I/R group, Emodin group, GRb1 group, and Emodin+GRb1 group had a significant difference at the time point of 6h, 1d, 3d, and 7d (*P* < 0.05, *P* < 0.01, or *P* < 0.001); Compared with GRb1 group, Emodin+GRb1 group had a significant difference at 1d and 3d (striatum) or 1d (cortex and hippocampus) after I/R (*P* < 0.05); Compared with Emodin group, Emodin+GRb1 group had a significant difference at 1d (hippocampus) or 1d and 3d (cortex and striatum) after I/R (*P* < 0.05 or *P* < 0.01); Compared with sham group, I/R group had a significant difference at 6h, 1d, 3d, and 7d (*P* < 0.001) (**Figure 7**).

## Discussion

The present study showed that the combination of GRb1 and Emodin has synergistically neuroprotective effects. The component compatibility of GRb1 and Emodin has relatively clear material basis and action mechanism, satisfying the quality control and neuroprotective function. Thus, the present study provides useful references on methods for the component compatibility and anti-stroke in further research. Furthermore, this study may provide the preclinical evidence for the combined use of GRb1 and Emodin on ischemic stroke, indicating that GRb1+Emodin can be a potential candidate for further clinical trials. Additionally, there are many other bioactive components of ginseng and Da Huang, such as Rb3, Rg1, Rd, and chrysophanol, whose component compatibility for cerebral ischemic stroke remains uncertain and are worth further research.

The highly recommended focal I/R injury model to imitate human ischemic stroke is transient suture-occluded MCAO method in rats (Zhou et al., 2014), of which the thread occlusion of the MCA modified by Longa et al. (1989) is well recognized worldwide. Thread occlusion of the MCA has well repeatability, causes less animal trauma and reduces animal mortality. The ischemic location adopted by this method is relatively fixed. Furthermore, thread can either be left in vessel for permanent occlusion or withdrawn to permit reperfusion, which is particularly suitable for researches of neuroprotection. Therefore, the present study adopted the modified thread occlusion of the MCA induced focal I/R injury model. The five-point neurologic grading scale using Zea-Longa criteria (Longa et al., 1989) was assessed at 2 h after ischemia in rats. Compared with sham group, I/R group had a significantly higher NDS and significantly larger infarction area after I/R, which demonstrated that the MCAO induced focal cerebral I/R injury model was successfully mimicked. In the present study, we also found that combination of Emodin and GRb1 had better and more stable effects on the functional recovery.

BBB is a dynamic interface between CNS and the peripheral blood circulation, playing a vital role in maintaining homeostasis in CNS (Obermeier et al., 2013; Liu and Su, 2014). Cerebral edema induced by the increasing permeability of the BBB can enlarge the infarction area in ischemic stroke (Zou et al., 2009; Shi et al., 2016). Thus, protecting BBB integrity and reducing the BBB permeability can effectively alleviate cerebral ischemic injury. Our results showed that the combination of Emodin and GRb1 has synergistic effects on reducing the BBB permeability and the infarction area.

Cerebral edema caused by cerebral ischemia can result in increased intracranial pressure and even cerebral hernia, leading to the death of ischemic stroke (Walberer et al., 2008). As the most highly expressed aquaporin in CNS, AQP4 plays an essential part in the formation and dissipation of cerebral edema (Nielsen et al., 1997; Papadopoulos and Verkman, 2007). Our previous study showed that basically consistent with the result of brain water content, AQP4 was upregulated and markedly expressed in the ipsilateral hemisphere after I/R (Lu et al., 2015). Aoki et al. (2003) found that the expression of AQP4mRNA significantly enhanced at the periphery of ischemic lesion with the exacerbation of cerebral edema in MCAO model. Thus, cerebral edema caused by cerebral ischemia may relate to the water transportation, in which AQP4 is involved, possibly resulting from the fact that the up-graduation of AQP4 make water transport rapidly from outside the cell and interstitial into cells. The present study demonstrated that monotherapy and combination therapy of Emodin and GRb1 have a neuroprotective effect via the down-regulation of AQP4, which may be associated with reduction of cerebral edema caused by ischemic stroke.

Gap junctions play an important role in intercellular communication and neuroprotection (Rana and Dringen, 2007). The function of GJ is related to the expression of Cx protein (Sahores and Mendoza-Naranjo, 2008). During I/R injury, as the function of GJ enhanced, Cx43 is considered to mediate spreading apoptosis information to adjacent injured cells, enlarging the infarction area (Nagy and Li, 2000; Belousov and Fontes, 2013). Frantseva et al. (2002) found that the function of GJ was enhanced in I/R injury and GJ inhibitors could alleviate I/R injury. Likewise, Cx43 specific inhibitor octyl alcohol can reduce the expression of Cx43 and infarction area by cutting off the communication between GJ, which results in neuroprotection (Niu et al., 2012). The present experimental results showed that monotherapy and combination therapy of Emodin and GRb1 have a neuroprotective effect via the down-regulation of Cx43.

Connexin 43 and AQP4 can interact in both structure and function. The expression of both Cx43 and AQP4 would decrease in the treatment of ischemic cerebral edema and the downregulation of AQP4 was also inhibited after applying Cx43 mimic peptide to inhibit Cx43 (Chu et al., 2017). Moreover, Cx43 and AQP4 could jointly participate in the transfer of K^+^ from astrocytes to blood vessels (Lichter-Konecki et al., 2008). And AQP4 may be integrated with Cx43 in astrocytic surface and potassium channel Kir4.1 to eliminate excess fluid (Wu et al., 2013). The present study mainly investigated the combined effect of Emodin and GRb1 on the neuroprotection in I/R injury, without research into the concentration-response relationship between AQP4 and Cx43 and whether there is interaction and its mechanism, which is the limitation of the present study as well as our further research direction.

## Conclusion

In conclusion, the combined use of Emodin and GRb1 could additively alleviate NDS, reduce the BBB permeability, reduce the infarction area and down-regulate Cx43 and AQP4 expression after I/R. Thus, the combination of Emodin and GRb1 exerted synergistically neuroprotective effects through regulating AQP4 and Cx43 expression after I/R.

## Author Contributions

YL and Q-qX contributed equally to this work. All authors contributed to writing of this manuscript.

## Conflict of Interest Statement

The authors declare that the research was conducted in the absence of any commercial or financial relationships that could be construed as a potential conflict of interest.

## References

[B1] Ahn S. M. Kim H. N. Kim Y. R. Choi Y. W. Kim C. M. Shin H. K. (2016). Emodin from *Polygonum multiflorum* ameliorates oxidative toxicity in HT22 cells and deficits in photothrombotic ischemia. *J. Ethnopharmacol.* 188 13–20. 10.1016/j.jep.2016.04.058 27151150

[B2] Aoki K. Uchihara T. Tsuchiya K. Nakamura A. Ikeda K. Wakayama Y. (2003). Enhanced expression of aquaporin 4 in human brain with infarction. *Acta Neuropathol.* 106 121–124. 10.1007/s00401-003-0709-y 12715185

[B3] Bangalore S. Schwamm L. Smith E. E. Singh I. M. Liang L. Fonarow G. C. (2014). Secondary prevention after ischemic stroke or transient ischemic attack. *Am. J. Med.* 127 728–738. 10.1016/j.amjmed.2014.03.011 24681258

[B4] Belousov A. B. Fontes J. D. (2013). Neuronal gap junctions: making and breaking connections during development and injury. *Trends Neurosci.* 36 227–236. 10.1016/j.tins.2012.11.001 23237660 PMC3609876

[B5] Belousov A. B. Fontes J. D. Freitas-Andrade M. Naus C. C. (2017). Gap junctions and hemichannels: communicating cell death in neurodevelopment and disease. *BMC Cell. Biol.* 18:4. 10.1186/s12860-016-0120-x 28124625 PMC5267333

[B6] Brouns R. De Deyn P. P. (2009). The complexity of neurobiological processes in acute ischemic stroke. *Clin. Neurol. Neurosurg.* 111 483–495. 10.1016/j.clineuro.2009.04.001 19446389

[B7] Chang Q. Pereda A. Pinter M. J. Balice-Gordon R. J. (2000). Nerve injury induces gap junctional coupling among axotomized adult motor neurons. *J. Neurosci.* 20 674–684. 10.1523/JNEUROSCI.20-02-00674.2000 10632597 PMC6772393

[B8] Chen W. Guo Y. Yang W. Zheng P. Zeng J. Tong W. (2015). Protective effect of ginsenoside Rb1 on integrity of blood-brain barrier following cerebral ischemia. *Exp. Brain Res.* 233 2823–2831. 10.1007/s00221-015-4352-3 26070903

[B9] Chu H. Huang C. Gao Z. Dong J. Tang Y. Dong Q. (2017). Reduction of ischemic brain edema by combined use of paeoniflorin and astragaloside IV via down-regulating connexin 43. *Phytother. Res.* 31 1410–1418. 10.1002/ptr.5868 28752625

[B10] Frantseva M. V. Kokarovtseva L. Perez Velazquez J. L. (2002). Ischemia-induced brain damage depends on specific gap-junctional coupling. *J. Cereb. Blood Flow Metab.* 22 453–462. 10.1097/00004647-200204000-00009 11919516

[B11] Gilleron J. Carette D. Segretain D. Pointis G. (2018). Multiple and complex influences of connexins and pannexins on cell death. *Biochim. Biophys. Acta* 1860 182–191. 10.1016/j.bbamem.2017.06.004 28625689

[B12] Gu Y. Zheng G. Xu M. Li Y. Chen X. Zhu W. (2012). Caveolin-1 regulates nitric oxide mediated matrix metalloproteinases activity and blood-brain barrier permeability in focal cerebral ischemia and reperfusion injury. *J. Neurochem.* 120 147–156. 10.1111/j.1471-4159.2011.07542.x 22007835

[B13] Han J. Y. Li Q. Ma Z. Z. Fan J. Y. (2017). Effects and mechanisms of compound Chinese medicine and major ingredients on microcirculatory dysfunction and organ injury induced by ischemia/reperfusion. *Pharmacol. Ther.* 177 146–173. 10.1016/j.pharmthera.2017.03.005 28322971

[B14] Jauch E. C. Saver J. L. Adams H. P. Bruno A. Connors J. J. Demaerschalk B. M. (2013). Guidelines for the early management of patients with acute ischemic stroke: a guideline for healthcare professionals from the American Heart Association/American Stroke Association. *Stroke* 44 870–947. 10.1161/STR.0b013e318284056a 23370205

[B15] Jeanson T. Pondaven A. Ezan P. Mouthon F. Charvériat M. Giaume C. (2016). Antidepressants impact connexin 43 channel functions in astrocytes. *Front. Cell. Neurosci.* 9:495. 10.3389/fncel.2015.00495 26778961 PMC4703821

[B16] Kong H. Fan Y. Xie J. Ding J. Sha L. Shi X. (2008). AQP4 knockout impairs proliferation, migration and neuronal differentiation of adult neural stem cells. *J. Cell. Sci.* 121 4029–4036. 10.1242/jcs.035758 19033383

[B17] Le H. T. Sin W. C. Lozinsky S. Bechberger J. Vega J. L. Guo X. Q. (2014). Gap junction intercellular communication mediated by connexin43 in astrocytes is essential for their resistance to oxidative stress. *J. Biol. Chem.* 289 1345–1354. 10.1074/jbc.M113.508390 24302722 PMC3894319

[B18] Li J. S. Liu J. X. Zhang W. Y. Liang S. W. Wang D. Fang J. (2005). Preventive effects of Emodin on cerebral ischemia injury and expression of the inflammatory factors in rats with cerebral ischemia. *China J. Chin. Mater. Med.* 30 1939–1943. 16494030

[B19] Lichter-Konecki U. Mangin J. M. Gordish-Dressman H. Hoffman E. P. Gallo V. (2008). Gene expression profiling of astrocytes from hyperammonemic mice reveals altered pathways for water and potassium homeostasis in vivo. *Glia* 56 365–377. 10.1002/glia.20624 18186079 PMC4116685

[B20] Liu A. J. Song L. Li Y. Zhang X. G. Chen Z. X. Huang L. B. (2015). Active compounds of rhubarb root and rhizome in animal model experiments of focal cerebral ischemia. *Evid. Based Complement. Alternat. Med.* 2015:210546. 10.1155/2015/210546 26495006 PMC4606211

[B21] Liu W. Y. Su D. F. (2014). Blood-brain barrier is not a barrier in the development of new drugs for ischemic stroke. *CNS Neurosci. Ther.* 20 1013–1014. 10.1111/cns.12356 25417927 PMC6493195

[B22] Longa E. Z. Weinstein P. R. Carlson S. Cummins R. (1989). Reversible middle cerebral artery occlusion without craniectomy in rats. *Stroke* 20 84–91. 10.1161/01.STR.20.1.84 2643202

[B23] Lu L. Li H. Q. Fu D. L. Zheng G. Q. Fan J. P. (2014). Rhubarb root and rhizome-based Chinese herbal prescriptions for acute ischemic stroke: a systematic review and meta-analysis. *Complement. Ther. Med.* 22 1060–1070. 10.1016/j.ctim.2014.10.002 25453529

[B24] Lu L. Li H. Q. Li J. H. Liu A. J. Zheng G. Q. (2015). Neuroprotection of sanhua decoction against focal cerebral ischemia/reperfusion injury in rats through a mechanism targeting aquaporin 4. *Evid. Based Complement. Alternat. Med.* 2015:584245. 10.1155/2015/584245 26089944 PMC4452182

[B25] Moussaddy A. Demchuk A. M. Hill M. D. (2018). Thrombolytic therapies for ischemic stroke: triumphs and future challenges. *Neuropharmacology* 134 272–279. 10.1016/j.neuropharm.2017.11.010 29505787

[B26] Mozaffarian D. Benjamin E. J. Go A. S. Arnett D. K. Blaha M. J. Cushman M. (2016). Heart disease and stroke statistics-2016 update: a report from the American Heart Association. *Circulation* 133 e38–e360. 10.1161/CIR.0000000000000350 26673558

[B27] Murthy H. N. Georgiev M. I. Kim Y. S. Jeong C. S. Kim S. J. Park S. Y. (2014). Ginsenosides: prospective for sustainable biotechnological production. *Appl. Microbiol. Biotechnol.* 98 6243–6254. 10.1007/s00253-014-5801-9 24859520

[B28] Nagy J. I. Li W. E. (2000). A brain slice model for in vitro analyses of astrocytic gap junction and connexin43 regulation: actions of ischemia, glutamate and elevated potassium. *Eur. J. Neurosci.* 12 4567–4572. 10.1046/j.1460-9568.2000.01331.x 11122370

[B29] Neuhaus A. A. Couch Y. Hadley G. Buchan A. M. (2017). Neuroprotection in stroke: the importance of collaboration and reproducibility. *Brain* 140 2079–2092. 10.1093/brain/awx126 28641383

[B30] Nielsen S. Nagelhus E. A. Amiry-Moghaddam M. Bourque C. Agre P. Ottersen O. P. (1997). Specialized membrane domains for water transport in glial cells: high-resolution immunogold cytochemistry of aquaporin-4 in rat brain. *J. Neurosci.* 17 171–180. 10.1523/JNEUROSCI.17-01-00171.1997 8987746 PMC6793699

[B31] Niu X. S. Maimaiti M. Dang H. Zhang X. H. Wang M. Y. Sha J. (2012). Neuroprotective effect of Cx43 blocking agent octanol on ischemia-reperfusion in mouse brain (in Chinese). *J. Apoplexy Nerv. Dis.* 29 409–411.

[B32] Obermeier B. Daneman R. Ransohoff R. M. (2013). Development, maintenance and disruption of the blood-brain barrier. *Nat. Med.* 19 1584–1596. 10.1038/nm.3407 24309662 PMC4080800

[B33] Papadopoulos M. C. Verkman A. S. (2007). Aquaporin-4 and brain edema. *Pediatr. Nephrol.* 22 778–784. 10.1007/s00467-006-0411-0 17347837 PMC6904420

[B34] Papadopoulos M. C. Verkman A. S. (2013). Aquaporin water channels in the nervous system. *Nat. Rev. Neurosci.* 14 265–277. 10.1038/nrn3468 23481483 PMC3732112

[B35] Powers W. J. Rabinstein A. A. Ackerson T. Adeoye O. M. Bambakidis N. C. Becker K. (2018). 2018 guidelines for the early management of patients with acute ischemic stroke: a guideline for healthcare professionals from the American heart association/american stroke association. *Stroke* 49 e46–e110. 10.1161/STR.0000000000000158 29367334

[B36] Rana S. Dringen R. (2007). Gap junction hemichannel-mediated release of glutathione from cultured rat astrocytes. *Neurosci. Lett.* 415 45–48. 10.1016/j.neulet.2006.12.043 17222973

[B37] Rha J. H. Saver J. L. (2007). The impact of recanalization on ischemic stroke outcome: a meta-analysis. *Stroke* 38 967–973. 17272772 10.1161/01.STR.0000258112.14918.24

[B38] Sahores M. Mendoza-Naranjo A. (2008). Gap junctions as therapeutic targets in brain injury following hypoxia-ischemia. *Recent Pat. CNS Drug Discov.* 3 209–215. 10.2174/157488908786242452 18991810

[B39] Shi Y. Leak R. K. Keep R. F. Chen J. (2016). Translational stroke research on blood-brain barrier damage: challenges, perspectives, and goals. *Transl. Stroke Res.* 7 89–92. 10.1007/s12975-016-0447-9 26757714 PMC4808584

[B40] Shobha N. Buchan A. M. Hill M. D. Canadian Alteplase for Stroke Effectiveness Study (Cases) (2011). Thrombolysis at 3–4.5 hours after acute ischemic stroke onset—evidence from the Canadian Alteplase for Stroke Effectiveness Study registry. *Cerebrovasc. Dis.* 31 223–228. 10.1159/000321893 21178345

[B41] Söhl G. Willecke K. (2004). Gap junctions and the connexin protein family. *Cardiovasc. Res.* 62 228–232. 10.1016/j.cardiores.2003.11.013 15094343

[B42] Vaibhav R. Juan S. M. Sylvain D. (2014). Ginseng: a promising neuroprotective strategy in stroke. *Front. Cell. Neurosci.* 8:457 10.3389/fncel.2014.00457 PMC4299449 25653588

[B43] Walberer M. Ritschel N. Nedelmann M. Volk K. Mueller C. Tschernatsch M. (2008). Aggravation of infarct formation by brain swelling in a large territorial stroke: a target for neuroprotection? *J. Neurosurg.* 9 287–293. 10.3171/JNS/2008/109/8/0287 18671642

[B44] Wang S. Hu Y. Tan W. Wu X. Chen R. Cao J. (2012). Compatibility art of traditional Chinese medicine: from the perspective of herb pairs. *J. Ethnopharmacol.* 143 412–423. 10.1016/j.jep.2012.07.033 22871585

[B45] Wang S. Li M. Guo Y. Li C. Wu L. Zhou X. F. (2017). Effects of *Panax notoginseng* ginsenoside Rb1 on abnormal hippocampal microenvironment in rats. *J. Ethnopharmacol.* 202 138–146. 10.1016/j.jep.2017.01.005 28065779

[B46] Wu B. Liu M. Liu H. Li W. Tan S. Zhang S. (2007). Meta-analysis of traditional Chinese patent medicine for ischemic stroke. *Stroke* 38 1973–1979. 10.1161/STROKEAHA.106.473165 17463317

[B47] Wu Z. Xu H. He Y. Yang G. Liao C. Gao W. (2013). Antisense oligodeoxynucleotides targeting connexin43 reduce cerebral astrocytosis and edema in a rat model of traumatic brain injury. *Neurol. Res.* 35 255–262. 10.1179/1743132813Y.0000000165 23485053

[B48] Wu Z. F. Luo C. X. Sun J. Han L. (2009). Effects of emodin on gene expression of NF-κB and ICAM-1 in cerebral tissue of rats with ischemic stroke. *J. Emerg. Tradit. Chin.* 18 934–936.

[B49] Yan Y. T. Li S. D. Li C. Xiong Y. X. Lu X. H. Zhou X. F. (2018). Panax notoginsenoside saponins Rb1 regulates the expressions of Akt/mTOR/PTEN signals in the hippocampus after focal cerebral ischemia in rats. *Behav. Brain Res.* 345 83–92. 10.1016/j.bbr.2018.02.037 29501622

[B50] Yao X. Derugin N. Manley G. T. Verkman A. S. (2015a). Reduced brain edema and infarct volume in aquaporin-4 deficient mice after transient focal cerebral ischemia. *Neurosci. Lett.* 584 368–372. 10.1016/j.neulet.2014.10.040 25449874 PMC4737527

[B51] Yao X. Uchida K. Papadopoulos M. C. Zador Z. Manley G. T. Verkman A. S. (2015b). Mildly reduced brain swelling and improved neurological outcome in aquaporin-4 knockout mice following controlled cortical impact brain injury. *J. Neurotrauma* 32 1458–1464. 10.1089/neu.2014.3675 25790314 PMC4589265

[B52] Zador Z. Bloch O. Yao X. Manley G. T. (2007). Aquaporins: role in cerebral edema and brain water balance. *Prog. Brain Res.* 161 185–194. 10.1016/S0079-6123(06)61012-1 17618977

[B53] Zhou Y. Li H. Q. Lu L. Fu D. L. Liu A. J. Li J. H. (2014). Ginsenoside Rg1 provides neuroprotection against blood brain barrier disruption and neurological injury in a rat model of cerebral ischemia/reperfusion through downregulation of aquaporin 4 expression. *Phytomedicine* 21 998–1003. 10.1016/j.phymed.2013.12.005 24462216

[B54] Zou W. Sun X. W. Yu X. P. Luo Y. M. Cheng H. K. Wang G. (2009). Research progress on blood-brain barrier and cerebral ischemia-reperfusion injury. *Chin. Arch. Tradit. Chin. Med.* 27 466–468. 10.13193/j.archtcm.2009.03.19.zouw.065

